# Expression of concern: Insect metamorphosis is regulated differently between sexes by members of a microRNA cluster

**DOI:** 10.7554/eLife.113553

**Published:** 2026-11-04

**Authors:** Timothy E Behrens

Li Chade, Chan Ki Kei, Nong Wenyan, Chen ShanShan, So Wai Lok, Qu Zhe, Wu Heidi YC, Yip Ho Yin, Chan Chi Bun, Tobe Stephen S, Bendena William G, Kai Zhen Peng, Hui Jerome HL. 2024. Insect metamorphosis is regulated differently between sexes by members of a microRNA cluster. eLife **13**:RP100499. doi: 10.7554/elife.100499.

Published 2 September 2024

The Editors are issuing this Expression of Concern to alert readers to issues relating to the methodology used for animal staging, which may affect the interpretation and validity of some of the study’s results. Readers are therefore advised to interpret the findings with caution. The concerns arose from the authors' responses to the original peer reviews.

1) Unexpected hormone levels and Kr-h1 expression

First, the reviewers raised concerns about unexpected results presented in the manuscript, including (a) the statement that "the levels of these hormones in female L3 larvae are equal to or greater than that seen at pupariation" and (b) the observation that peak Kr-h1 expression occurs in L3 larvae, when juvenile hormone (JH) titres would be expected to be low, followed by a decrease during wandering. The reviewer noted that this pattern differs from that reported previously, including in FlyBase and modENCODE.

In response, the authors suggested that differences in developmental staging, methodology, normalisation, strain and/or experimental conditions could account for the discrepancies. However, the authors did not provide an explanation that resolved the Editors' concerns about the unexpected hormone levels and Kr-h1 expression pattern.

2) Criteria used to define developmental stages

Second, the reviewers were concerned about the criteria used to define the developmental stages of the animals used in the experiments.

In response to a reviewer’s request for greater precision in describing the developmental stages, the authors stated that the "L3 larvae" or "larva" referred specifically to wandering third-instar larvae (WL3) that had exited the food, had approximately one-third to one-quarter of their gut contents remaining, and displayed protruding anterior spiracles. However, protrusion of the anterior spiracles is associated with a relatively brief period during the wandering stage and may therefore be difficult to use as the criteria to define developmental stage of animals. In addition, the images provided for the wandering L3 larvae did not appear to correspond to the stage described by the authors. The authors then go on to describe WL3 larvae as individuals that had completely exited the food and were climbing upwards in the fly vials, which is inconsistent with their prior responses.

The editors subsequently asked the authors to clarify how the experimental samples had been staged. The authors clarified that the experimental samples were not restricted to a precisely defined developmental stage.

This lack of a clearly defined and consistently applied staging criterion is of concern because hormone titres and gene expression can change substantially during the transition from late L3 larvae through wandering larvae and into pupariation. Consequently, developmental heterogeneity within the experimental samples could affect the reported hormone measurements and gene expression profiles, and may contribute to some of the unexpected findings described above.

3) Interpretation of the effects of miR-277/34 on target genes

Third, the editors note that, although microRNAs generally act to repress the expression of target genes, the manuscript proposed a model in which miR-277/34 activate the expression of two specific target genes, without providing sufficient experimental evidence to support such a mechanism.

In response, the authors clarified that the larvae used for the real-time PCR experiments presented in Figures 1 and 2 were described as Stages I and II, and acknowledged that these samples may have comprised a mixture of animals at different developmental stages. The authors also clarified that they did not intend to conclude that miR-277 or miR-34 directly activates these genes.

This clarification does not, however, resolve the concerns regarding the interpretation of the gene-expression results, particularly given the uncertainty surrounding the developmental staging of the experimental samples.

